# Uterine artery Doppler study in second trimester of pregnancy

**DOI:** 10.11604/pamj.2013.15.87.2321

**Published:** 2013-07-05

**Authors:** Olufemi Adebari Oloyede, Faye Iketubosin

**Affiliations:** 1 Fetal Medicine Unit, Department of Obstetrics & Gynaecology, Lagos State University Teaching Hospital, Ikeja, Lagos, Nigeria; 2 Assisted Conception Unit, Georges Memorial Medical Centre, Lagos, Nigeria

**Keywords:** Uterine artery Doppler, pulsatility index, resistance index, systolic/diastolic ratio, impaired placentation

## Abstract

**Introduction:**

The uterine artery Doppler has potentials for screening for complications of impaired placentation. This study examines the indices of uterine artery impedance at 22-23 weeks gestation and their relationship with maternal age and parity.

**Methods:**

Uterine artery colour imaging and pulsed wave Doppler ultrasound was conducted between 22nd and 23rd weeks in 430 pregnancies. The pregnancies were classified into 2 groups: normal and abnormal outcomes. The indices of impedance recorded were pulsatility index, resistance index and the systolic/diastolic ratio. Relevant obstetric information was retrieved from the antenatal records. The student t- test and Pearson's product moment were used for statistical analysis.

**Results:**

Fifty eight (13.5%) out of 430 pregnancies had complications of impaired placentation, mainly intrauterine growth restriction and preterm birth (24 or 41.4% each). The indices in normal pregnancies were similar to presently used values. There was no statistically significant difference in the 2 pregnancies groups. The Pulsatility Index (PI) in the right uterine artery was statistically different from the left (t-test 32.8, p < 0.05). Maternal age and parity demonstrate statistically significant positive correlation with PI (r =0.9, p < 0.05; r =0.8, p < 0.05).

**Conclusion:**

The indices in normal pregnancies were similar to values from previous studies. The values are however not significantly different in pregnancies with abnormal outcome.

## Introduction

The complications of impaired placentation are significant contributors to maternal and perinatal morbidity and mortality in both developing and developed countries [1–3]. A large number of researches have investigated the potentials of second trimester uterine artery Doppler studies as a screening tool for these complications [4–6]. Although research findings are not uniformly in favour of its use, because of the heterogeneity of the aetiology of complications of impaired placentation, there are however important evidences to justify the application of presently used indices for routine screening [4, 5].

Pregnancies affected by the complications of impaired placentation such as pregnancy induced hypertension, intra uterine fetal growth restriction and preterm birth have been shown to demonstrate increased impedance in the spiral artery [4]. The spiral artery, the major continuation of the uterine artery undergoes trophoblastic invasion during pregnancy. This physiological process is characterized by loss of the musculoelastic properties and its conversion to the uteroplacental arteries, which allows an increased blood flow to the placenta and the fetus. This process commences in the first and ends in early second trimester [7]. Second trimester Doppler is usually performed between 20th and 24th weeks of pregnancy, when it is expected that the physiologic process would have been completed.

The impairment or complete absence of the physiological process is associated with increased vascular resistance and increased impedance to blood flow and ultimately affect blood flow into the placenta [8]. These sequences of events precede the onset of the complications [9]. The effect of abnormal trophoblastic invasion is derived from studies on the uterine artery, because the uterine artery provides a good representation of the sum of resistances of the placental bed and of the placental perfusion [8, 10]. Doppler flow studies of the uterine artery therefore provides an accurate means of assessing uteroplacental resistance to blood flow and a good method of assessing impairment or absence of uteroplacental blood flow [10].

The uterine artery Doppler is justifiably recommended in developing countries because of the high prevalence of mortality and morbidity due to complications of impaired placentation.

The purpose of this study was to determine the range, mean and standard deviations of pulsatility index (PI), resistance index (RI) and systolic/diastolic (S/D) ratio of the uterine artery at 22nd & 23rd weeks of pregnancy with normal as well as those with abnormal outcome. It also determines the relationship between the indices and parity and maternal age.

## Methods

In a cross sectional study, 435 pregnant women had Doppler ultrasound examination of the uterine artery immediately after routine second trimester anomaly scan between 1st January, 2010 and 30th June, 2012. Approval for study was obtained from the management board of High Rocks Fetal Medicine and Genetic Diagnosis Centre, Lagos, where the procedure was done and Georges Memorial Hospital, Lagos, where the patients received antenatal and postnatal care. All the women provided informed written consent. The study was carried out by a trained Feto-Maternal Medicine Specialist, certified by the Fetal Medicine Foundation, London in the conduct of 18-23 weeks scan. All studies were performed between 22nd and 23rd weeks of pregnancy after routine anomaly scan done with Imagynae and HDI 1500 scanning machine using a 3.5-5.0MHz transabdominal sector transducer. The inclusion criteria were singleton pregnancy, absence of medical disorder in index pregnancy and gestational age between 22nd and 23rd weeks based on a first trimester ultrasound scan. Excluded were pregnancies from assisted conceptions techniques, plural pregnancy, underlying medical disorder or gynaecological history known to be associated with high risk for any of the complications, on-going treatment that may influence pregnancy outcome and failure to consent.

**Figure 1:**
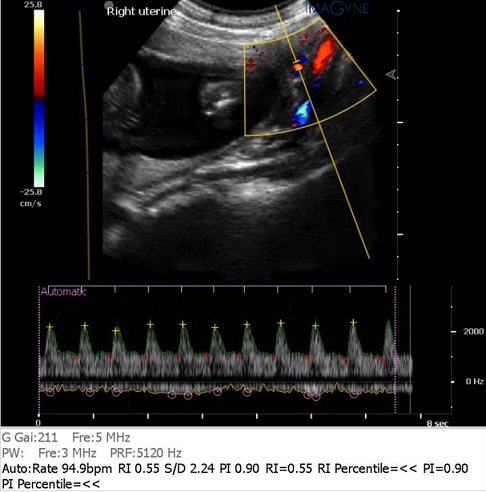
Doppler waveform/pattern

In semi recumbent position, the ultrasound transducer was placed in either the left or right iliac fossae of the abdomen, directed towards the lateral uterine walls and downwards into the pelvis, to obtain the sagittal section of the uterus and cervical canal. This is followed by the introduction of the colour flow imaging to produce a colour map of flow over the region. The probe is tilted sideways but still maintaining its medial angulation (lower paracervical area), till the uterine artery is visualized as it crosses the external iliac artery, having originated from the internal iliac artery. The sample volume was placed 1cm distal to the point of apparent cross over before any branching of the uterine arteries and the angle of insonation maintained below 500. These characters were used as the standard landmark for investigation of the uterine artery (Figure 1). Pulsed Doppler gate was placed at this location to obtain flow waveforms and when at least 3 consecutive consistent waveforms are produced, the image is frozen. The Doppler indices generated automatically from the machine were, the Pulsatility Index (PI), Resistance Index (RI) and S/D Ratio. Antenatal clinics were routinely conducted till delivery and the women followed up in the postnatal clinic. Outcome was classified into normal or abnormal. In the study, pregnancy is considered to have abnormal outcome, if it is complicated by any of the complications associated with impaired placentation. These include Pregnancy Induced Hypertension (PIH), Intra uterine growth restriction (IUGR) or Preterm birth. PIH was diagnosed by a rise in systolic pressure of at least 30 mm of Hg or a rise in diastolic pressure of at least 15 mm of Hg over the previously known blood pressure; an absolute rise in the blood pressure of at least 140/90 mm of Hg; diastolic blood pressure > 90 mmHg measured on at least two occasions after 20 weeks of gestation in a previously normotensive woman (International Society for the Study of Hypertension in Pregnancy (ISSHP). Intrauterine growth restriction is defined by fetal weight below the 10th percentile of the average weight for gestational age [11]. Preterm birth refers to the delivery of the baby before 37th week of pregnancy [3]. In the absence of any of these conditions, the pregnancy is classified as one with normal outcome.

Records of events during pregnancy and post natal period were retrieved from the clinical case notes. The indices obtained in pregnancies with normal outcome were classified as normal ranges of Doppler indices Demographic characteristics of the women were retrieved from unit data base. The range, mean and standard deviations of the indices from both right and left arteries were analysed. Student's t test was used to compare means values between normal and abnormal pregnancy outcome, Pearson?s Product Moment was used to determine correlation between variables. Statistical significance was set at p< 0.05.

## Results

Figure 1 shows the site of insonation of the uterine artery used for interrogation. Uterine artery Doppler study was conducted on 430 pregnant women that consented to participate in the study. Of these, 372 (86.5%) pregnancies had normal outcome, while 58 (13.5%) pregnancies had abnormal outcome (Complications of impaired placentation).

The Doppler indices in the women that had normal pregnancy outcome are shown in Table 1. The mean PI in the right and left uterine artery are 1.09 and 0.81, with a range of 0.53 - 1.58 and 0.58 - 1.83 respectively. The RI has a mean of 0.59 and 0.65, while the range was 0.37-1.16 and 0.41 - 0.82 in both the right and left uterine artery respectively. The mean, S/D ratio was 2.56 and 2.57 respectively and range 1.53 - 3.90 and 1.92 - 3.04 respectively in the right and left uterine artery. The difference between the right and left uterine PI was statistically significant (t-32.8, p < 05).

**Table 1:** Uterine Artery Indices in Pregnancies with normal outcome

Indices		Range	Mean		Significance (t-test)
	RT	LT	RT	LT	
PI	0.53-1.58	0.58-1.83	1.09 ± 0.55	0.81 ± 0.53	32.81 (p < 0.05) S
RI	0.37-1.16	0.41-0.82	0.59 ± 0.13	0.65 ± 0.15	1.67 (p > 0.05) NS
S/D	1.53-3.90	1.92–3.04	2.56 ± 0.53	2.57 ± 0.5	40.17 (p < 0.05) NS

S = significant difference NS = No significant difference

Complications were recorded in 58 (13.5%) pregnancies. Of there, preterm birth and IUGR were the most common (24 or 41.4% each). Pregnancy induced hypertension (PIH) was recorded in 10 (17.2) pregnancies (Table 2).

**Table 2:** Pregnancy Outcome

Outcome	Number	Percentage (%)
Normal	372	86.5
**Abnormal (complications)**	**58**	**13.5**
Hypertension	10	2.2
Preterm birth	24	5.5
IUGR	24	5.5

Table 3 shows that the mean indices are generally higher in pregnancies with abnormal outcome compared with pregnancies with normal outcome. The difference is however not statistically significant. The t- test for the PI, RI and S/D ratio were 0.08 (p > 0.05), 0.07 (p > 0.05) and 0.06 (p > 0.05) respectively.

**Table 3:** Uterine Artery Indices in pregnancies with Normal and Abnormal Outcome

Indices	Normal Outcome	Abnormal Outcome		
	Mean (n = 372)	Mean (n = 63)	P Value	t -Test
PI	0.95	1.20	> 0.05	0.08 (NS)
RI	0.62	0 .63	> 0.05	0.07 (NS)
S/D	2.41	2.52	> 0.05	0.06 (NS)

S = significant difference NS = No significant difference

Maternal age correlated strongly with PI (r =1.12, p 0.05), but weakly with RI (r =0.37, p 0.05). There was also a statistically significant correlation between the PI and RI as maternal age advances (r = 0.78, p 0.05). Parity has a strong positive correlation with PI (r = 0.81, p 0.05) and a slight positive correlation with RI (r = 0.63, p 0.05). The correlation between PI and RI along parity was also slightly positive (r = 0.38, p 0.05) (Table 4)

**Table 4:** Relationship between Uterine Artery Indices and Obstetric variables

Characteristic	Number (n = 435)	Mean PI	Mean RI	Mean S/D
**Age**				
< 25	24	0.98	0.56	2.21
26-30	221	1.05	0.59	3.00
31-35	95	1.05	0.67	3.04
36-40	71	1.09	0.68	2.47
> 40	24	1.31	0.70	2.21
{MA and PI (r =0.9, p < 0.05); MA and RI (r =0.4, p > 0.05); PI and RI (r = 0.8, p > 0.05)}			
**Parity**				
0	115	1.02	0.60	3.03
1	109	1.31	0.67	3.20
2	101	1.39	0.69	3.32
3	67	1.41	0.72	3.38
4	43	1.38	0.70	3.21
{Parity and PI (r = 0.8, p < 0.05); Parity and RI (r = 0.6, p > 0.05); PI and RI (r = 0.4, p > 0.05)}			

## Discussion

The Doppler indices obtained from the study in pregnancies with normal outcome were similar to values in previous study and also similar to values in current use in many centres [8]. The study also shows that there was no statistical difference between the values in pregnancies with normal and those with abnormal outcome. The apparent implication of this is that the uterine artery Doppler may not have a role in the screening for pregnancies with complications of impaired placentation. This however contradicts other studies where it has been recommended for this purpose [10]. In practical terms, our conclusion may have been affected by some factors. It is possible that the small sample size could have a significant effect on this conclusion and a larger sample size would be needed to firmly establish the observation. Trio factors namely, low utilization of orthodox antenatal care services, scarcity and paucity of use Doppler scan in antenatal management in many developing centres and low socioeconomic status are recognized factors for small sample size. A suggestion to improve on the small sample size is the introduction of Doppler ultrasound scan as part of routine anomaly scan. This is because of the widespread utilization of scan during the second trimester by many health workers and pregnant women. [12]. We took advantage of the second trimester anomaly scan to conduct uterine artery screening at same session. In addition to the factors discussed above, another potential reason for the absence of difference in indices between normal and abnormal pregnancies relates to the heterogeneity of aetiologies of the complications, which is not usually considered in many studies design [10]. This also contributes to the apparent poor sensitivity and specificity in some studies [5, 10].

The complications recorded in the study were similar to reports from other studies [8, 10]. It is however striking that hypertensive disorders was not the most common in the study contrary to reports from other studies [5, 8, 10]. This could be an indication that the cases of hypertensive disorders seen in the locality may not be due mainly to impaired placentation. Consequently, there would be no difference in the uterine indices compared with normal pregnancies. Maternal disorders such as diabetes mellitus, coagulation disorders e.t.c., are reported to be major contributors to this group of hypertensive disorders, which are characteristically late onset and less severe [10]. Intrauterine growth restriction was one of the duo commonest complications. It is speculated that reduced uteroplacental perfusion due to impaired placentation may occur before 20 weeks and caused IUGR, despite a restoration of uteroplacental flow and normal Doppler indices at 22nd to 23rd week when screening was done [5, 8].

In the present study, preterm birth was one of the commonest complications. However, we found no difference in the Doppler indices between pregnancies that are complicated by preterm birth and term deliveries. This is contrary to another study that reported an association between preterm birth and abnormal Doppler indices [10]. It is possible that majority of these cases were due to therapeutic intervention following other complications especially, hypertensive disorder.

The influence of maternal age and parity on obstetric measurements has been emphasized by the result of the study. Increasing maternal age has an independent association with specific adverse outcomes in pregnancy [13]. The implication of our observation is that interpretation of Doppler indices has to consider these variables namely, maternal age and parity in order to achieve the best results and uniformity of outcomes form different studies.

The result of this study is suitable for clinical application, because three important factors were considered. First, all procedures were done in the 22nd - 23rd week of pregnancy, when the process of physiological adaptation of the spiral arteries would have been completed [10]. Secondly, the proximal part of the uterine artery where it crosses the external iliac was chosen as insonation site as recommended by other workers [5, 14]. At this point, the uterine artery reflects the total impedance to flow in the distal uteroplacental circulation supplied by the spiral or arcuate arteries [8, 10]. Thirdly, only one trained and experienced operator performed all procedures to reduce inter-observer and intra-observer coefficients of variation which could be as high as 10.1% and 10.8% for PI [10]. We are however aware that abnormal physiological adaptation could in some instances have occurred and normalized before 20 weeks, while 24 weeks is generally considered too late a time to commence intervention in severe cases based on an abnormal findings [5, 15]. We recommend the 22nd - 23rd week for 2 reasons. First, majority of those with earlier onset abnormal physiological adaptation would still persist till this period and second, those who have late abnormality of physiological adaptation would also be identified [5]. The low predictive and sensitivity profile of uterine Doppler due to poor understanding of the heterogeneity of aetiology factors as well as economic viewpoint precludes its recommendation for routine use. Future researches should focus more on methods to standardize indications, addition of biochemical and clinical markers and possibility of late first trimester screening. The benefit would be an earlier commencement of preventive measures such as low dose aspirin in at risks women and an overall better pregnancy outcome.

## Conclusion

This study establishes the Doppler indices in pregnancies with normal outcome, which is recommended for clinical use in the locality. It also recommends the interpretation in relation to maternal age and parity. Further study is required to confirm the role in screening for risk of complications of impaired placentation in the second trimester.
